# A phase Ib study of pictilisib (GDC-0941) in combination with paclitaxel, with and without bevacizumab or trastuzumab, and with letrozole in advanced breast cancer

**DOI:** 10.1186/s13058-018-1015-x

**Published:** 2018-09-05

**Authors:** Patrick Schöffski, Sara Cresta, Ingrid A. Mayer, Hans Wildiers, Silvia Damian, Steven Gendreau, Isabelle Rooney, Kari M. Morrissey, Jill M. Spoerke, Vivian W. Ng, Stina M. Singel, Eric Winer

**Affiliations:** 1 Department of General Medical Oncology, Leuven Cancer Institute, University Hospitals Leuven, Leuven, Belgium; 2 Department of Oncology, Faculty of Medicine, Laboratory of Experimental Oncology, KU Leuven, Herestraat 49, B-3000 Leuven, Belgium; 3 Department of Medical Oncology, Fondazione IRCCS Istituto Nazionale dei Tumori, Milan, Italy; 4 Department of Medicine, Vanderbilt University Medical Center, Nashville, TN USA; 5 Oncology Biomarker Development, Genentech Inc, South San Francisco, CA USA; 6 Product Development Oncology, Genentech Inc, South San Francisco, CA USA; 7 Clinical Pharmacology, Genentech Inc, South San Francisco, CA USA; 8 Biostatistics, Genentech Inc, South San Francisco, CA USA; 9 Department of Medical Oncology, Dana-Farber Cancer Institute, Boston, MA USA

**Keywords:** Pictilisib, GDC-0941, PI3K

## Abstract

**Background:**

This phase Ib study (NCT00960960) evaluated pictilisib (GDC-0941; pan-phosphatidylinositol 3-kinase inhibitor) plus paclitaxel, with and without bevacizumab or trastuzumab, or in combination with letrozole, in patients with locally recurrent or metastatic breast cancer.

**Methods:**

This was a three-part multischedule study. Patients in parts 1 and 2, which comprised 3 + 3 dose escalation and cohort expansion stages, received pictilisib (60–330 mg) plus paclitaxel (90 mg/m^2^) with and without bevacizumab (10 mg/kg) or trastuzumab (2–4 mg/kg). In part 3, patients received pictilisib (260 mg) plus letrozole (2.5 mg). Primary objectives were evaluation of safety and tolerability, identification of dose-limiting toxicities (DLTs) and the maximum tolerated dose (MTD) of pictilisib, and recommendation of a phase II dosing regimen. Secondary endpoints included pharmacokinetics and preliminary antitumor activity.

**Results:**

Sixty-nine patients were enrolled; all experienced at least one adverse event (AE). Grade ≥ 3 AEs, serious AEs, and AEs leading to death were reported in 50 (72.5%), 21 (30.4%), and 2 (2.9%) patients, respectively. Six (8.7%) patients reported a DLT, and the MTD and recommended phase II pictilisib doses were established where possible. There was no pictilisib–paclitaxel drug–drug interaction. Two (3.4%) patients experienced complete responses, and 17 (29.3%) patients had partial responses.

**Conclusions:**

Combining pictilisib with paclitaxel, with and without bevacizumab or trastuzumab, or letrozole, had a manageable safety profile in patients with locally recurrent or metastatic breast cancer. The combination had antitumor activity, and the additive effect of pictilisib supported further investigation in a randomized study.

**Trial registration:**

ClinicalTrials.gov, NCT00960960. Registered on August 13, 2009.

**Electronic supplementary material:**

The online version of this article (10.1186/s13058-018-1015-x) contains supplementary material, which is available to authorized users.

## Background

Despite improvements in treatment outcomes for patients with metastatic breast cancer, there is a continued unmet need to improve therapies for this patient population. Breast cancer is a heterogeneous disease, and current treatment strategies are based on disease type. Hormone receptor-positive, recurrent, or stage IV breast cancer in postmenopausal women is often managed with endocrine therapies, such as the nonsteroidal aromatase inhibitor letrozole [1], whereas primary treatment options for patients with human epidermal growth factor receptor 2 (HER2)-negative locally recurrent or metastatic breast cancer include single-agent cytotoxic chemotherapeutic agents, such as paclitaxel [1, 2]. Addition of the monoclonal antibody bevacizumab, which blocks angiogenesis by inhibiting vascular endothelial growth factor A, to paclitaxel has been shown to improve progression-free survival (PFS) and objective response rate (ORR) in patients with first-line metastatic breast cancer [1, 3]. In patients with HER2-positive locally recurrent or metastatic breast cancer, therapies include the antibody–drug conjugate ado-trastuzumab emtansine and the combination of the monoclonal antibodies trastuzumab and pertuzumab, both of which target HER2, with either docetaxel or paclitaxel [1, 2].

The phosphatidylinositol 3-kinase (PI3K) signaling pathway is deregulated in a wide variety of cancers, including breast cancer [4–6], and plays a key role in cell growth, survival, and migration [7]. The PI3K lipid kinases are grouped according to substrate specificity, structure, and mechanism of action into three classes (IA, IB, II, and III) [8]. Activating mutations of the catalytic subunit of PI3K (phosphatidylinositol-4,5-bisphosphate 3-kinase catalytic subunit alpha [*PIK3CA*]), which belongs to the class IA PI3K family, are frequently observed in breast cancer [9, 10], and approximately 35–45% of cases of hormone receptor-positive breast cancer harbor mutations in this gene [11, 12]. Preclinical data suggest that activation of the PI3K pathway, via mutation of *PIK3CA*, loss of phosphatase and tensin homolog (PTEN) expression, or HER2 overexpression, may promote resistance to antiestrogen therapy and hormonal independence in estrogen receptor (ER)-positive models of breast cancer [13–15]. In addition, results from three clinical trials suggest that inhibition of both the PI3K/mammalian target of rapamycin (mTOR) and estrogen-signaling pathways may provide improved efficacy compared with single-agent endocrine therapies [16–18]. Thus, inhibition of the PI3K pathway has emerged as a promising strategy for treatment of breast cancer.

Pictilisib (GDC-0941) is a potent and selective oral inhibitor of class I PI3K [19] that prevents the formation of phosphatidylinositol (3,4,5)-trisphosphate, a key component of the PI3K pathway, by binding to the adenosine triphosphate-binding pocket of PI3K [19]. Pictilisib is a pan-PI3K inhibitor that inhibits all four isoforms of class I PI3Ks (p110α, p110β, p110δ, and p110γ subunits) [19] with rapid absorption following oral administration and a dose-proportional pharmacokinetic (PK) profile [20]. Pan-PI3K inhibitors may be better suited to combination therapy than inhibitors of mammalian target of rapamycin complex 1/2, and there is evidence that their activity may not be restricted to tumor types with *PIK3CA* mutations [21]. In contrast, isoform-specific PI3K inhibitors such as alpelisib (BYL719) and taselisib (GDC-0032), which both selectively target PI3Kα [22, 23], offer the potential specifically to block their target while limiting toxicities associated with a broader inhibition [21].

In preclinical studies, pictilisib had antitumor activity in breast cancer models harboring *PIK3CA* mutations and/or amplification of HER2, although several models without these mutations were also sensitive to pictilisib treatment [24]. Pictilisib was found to increase the antitumor activity of taxanes, with an associated increase in apoptotic cell death, in multiple breast cancer xenografts [25] and, in combination with trastuzumab, synergistically inhibited cell proliferation and the PI3K signaling pathway in HER2-amplified breast cancer cell lines [26]. Pictilisib was also reported to inhibit the growth of activated human endothelial cells, suggesting the potential for antiangiogenic activity [27].

Single-agent pictilisib was well tolerated and showed evidence of antitumor activity in a phase I study of 60 patients with solid tumors at doses ≥ 100 mg [20]. In addition, the PK profile of single-agent pictilisib was dose-proportional, with a maximum tolerated dose (MTD) of 330 mg administered orally daily [20]. Several studies have investigated the effect of PI3K inhibition in patients with breast cancer and alterations of the PI3K pathway. The phase III BOLERO-2 trial demonstrated that the mTOR inhibitor everolimus, when combined with an aromatase inhibitor, improved PFS in hormone receptor-positive advanced breast cancer previously treated with nonsteroidal aromatase inhibitors [17], although there was no statistically significant improvement in overall survival [28].

This open-label, multischedule phase Ib study aimed to evaluate the safety and PK of pictilisib in combination with paclitaxel, with and without bevacizumab or trastuzumab, or letrozole, in patients with locally recurrent or metastatic breast cancer. In addition, we sought to establish a recommended phase II dose for each treatment combination regimen.

## Methods

### Patients

Eligible patients were ≥ 18 years with histologically or cytologically confirmed locally recurrent or metastatic adenocarcinoma of the breast. Inclusion criteria specified that patients had HER2-negative tumors, unless in the cohort that received trastuzumab, where all patients were required to have HER2-positive tumors and an Eastern Cooperative Oncology Group Performance Status (ECOG PS) of 0 or 1. Patients who received letrozole were postmenopausal and required to have hormone receptor-positive disease. Adequate hematologic and end-organ function was required, in addition to disease measurable by Response Evaluation Criteria In Solid Tumors (RECIST) v1.0.

Patients who had received more than two prior chemotherapy regimens for locally recurrent or metastatic breast cancer were not eligible for inclusion in the arms that received pictilisib + paclitaxel ± bevacizumab or trastuzumab treatment (parts 1 and 2). Patients were eligible for enrollment in the pictilisib + letrozole arm (part 3) if they were currently receiving letrozole for the treatment of advanced or metastatic breast cancer, but they were excluded if they had received more than one prior chemotherapy regimen or more than two prior endocrine therapy regimens for locally recurrent or metastatic breast cancer. Patients with known hypersensitivity to paclitaxel were excluded.

Patients were not eligible for bevacizumab treatment if they had inadequately controlled hypertension, significant vascular disease within 6 months prior to the first dose of study treatment, history of hemoptysis within 1 month prior to the first dose of study treatment, or evidence of bleeding diathesis or significant coagulopathy. Patients were not eligible for trastuzumab treatment if they had a history of grade ≥ 3 hypersensitivity to the antibody, or grade ≥ 1 with the most recent trastuzumab infusion before study entry, or continued requirement for prolonged trastuzumab infusions (> 30 minutes) to prevent infusion-related reactions. Patients with a history of exposure to anthracyclines (cumulative doses > 500 mg doxorubicin, > 900 mg liposomal doxorubicin, > 900 mg epirubicin, > 120 mg mitoxantrone, and > 90 mg idarubicin; if another anthracycline or more than one anthracycline was used, the cumulative dose could not exceed the equivalent of 500 mg doxorubicin) and cardiopulmonary dysfunction were also excluded.

### Study design and treatment

This was an open-label, multicenter, phase Ib dose escalation study (ClinicalTrials.gov registration number NCT00960960) performed in three parts. Parts 1 and 2 comprised two stages (a 3 + 3 dose escalation stage and a cohort expansion stage), with the dose escalation stage designed to evaluate the safety, tolerability, and PK of pictilisib in combination with paclitaxel, or with paclitaxel plus bevacizumab or trastuzumab. Part 3 had a 3 + 3 dose escalation enrollment design and assessed the combination of pictilisib and letrozole. Patients were assigned in the order in which they were enrolled.

In part 1, patients received oral pictilisib (at an initial dose of 60 mg) administered daily on days 1–21 of each 28-day cycle (“21 + 7” schedule) and 90 mg/m^2^ intravenous paclitaxel (cohort 1) or 90 mg/m^2^ intravenous paclitaxel plus 10 mg/kg intravenous bevacizumab (all subsequent cohorts). On study treatment days, pictilisib was administered prior to paclitaxel or bevacizumab. Paclitaxel was administered on days 1, 8, and 15 of each 28-day cycle, and bevacizumab was administered on days 1 and 15 of each 28-day cycle.

In part 2, patients received oral pictilisib (daily for 5 of 7 consecutive days [“5 + 2” schedule]) in combination with 90 mg/m^2^ intravenous paclitaxel. Once the MTD had been established, two additional arms were opened to determine the MTD for pictilisib in combination with paclitaxel plus 10 mg/kg intravenous bevacizumab or 2–4 mg/kg intravenous trastuzumab. The starting dose for pictilisib in combination with paclitaxel plus bevacizumab was at or below the MTD for pictilisib plus paclitaxel, whereas the starting dose for pictilisib in combination with paclitaxel plus trastuzumab was at least one dose level below the MTD for pictilisib plus paclitaxel alone. Paclitaxel was administered on days 1, 8, and 15 of each 28-day cycle; bevacizumab was administered on days 1 and 15 of each 28-day cycle; and trastuzumab was administered on days 1, 8, 15, and 22 of each 28-day cycle.

In part 3, patients were treated with 260 mg pictilisib plus 2.5 mg letrozole by continuous daily dosing in 28-day cycles.

Either the MTD or a lower dose was selected as the recommended phase II dose. This was dependent on both the MTD-defining dose-limiting toxicities (DLTs) and the adverse events (AEs) reported during the DLT observation period and beyond in all patients treated at a given dose. Study treatment was discontinued in patients who experienced disease progression or unacceptable toxicity or who were not compliant with the study protocol.

Tumor assessments were performed according to RECIST v1.0. In parts 1 and 2, assessments were performed at screening and at the end of cycles 2, 5, 8, and 11 and every three cycles thereafter for patients who were on the study for > 1 year. Objective responses were confirmed ≥ 4 weeks after the initial documentation. In part 3, assessments were performed at screening and on day 1 of cycle 4 (± 7 days) and on day 1 (± 7 days) every three cycles thereafter.

### Safety assessment

AEs were graded according to the National Cancer Institute Common Terminology Criteria for Adverse Events, version 3.0 [29]. AEs were recorded until 30 days after the last dose of study treatment or until initiation of another anticancer therapy, whichever occurred first.

DLT was defined as one of the following AEs, if occurring during the DLT assessment window (following the first dose of pictilisib and including evaluations prior to dosing on day 1 of cycle 2) and considered by the investigator to be related to study treatment: grade ≥ 3 nonhematologic, nonhepatic major organ AE; grade ≥ 4 thrombocytopenia lasting > 48 hours or associated with clinically significant bleeding; grade ≥ 3 fasting hyperglycemia; grade ≥ 4 neutropenia lasting ≥ 7 days; grade ≥ 3 febrile neutropenia; grade ≥ 3 total bilirubin, alkaline phosphatase (ALP), or hepatic transaminases (alanine aminotransferase or aspartate aminotransferase); grade ≥ 2 lung diffusing capacity concomitant with a decrease of ≥ 20% from baseline.

AEs that were not considered DLTs included grade 3 nausea, vomiting, or diarrhea that resolved to grade ≤ 1 with optimal medical management within 3 days, grade 3 hypertension for patients receiving bevacizumab, grade 3 fasting hyperglycemia that resolved to grade ≤ 1 within 7 days (with or without antihyperglycemic therapy), and grade 3 fasting hyperglycemia within 3 days of glucocorticoid use. For patients with grade 1 hepatic transaminase levels at baseline, a hepatic transaminase elevation > 7.5 times the upper limit of normal (ULN) was considered a DLT. For patients with grade 1 ALP levels at baseline, an elevation > 7.5 times the ULN was considered a DLT.

The DLT assessment window followed the first dose of pictilisib and included evaluations prior to dosing on day 1 of cycle 2. The MTD was exceeded if a DLT was observed in at least one-third of patients or if greater than one-third of patients in a cohort missed ≥ 5 days of pictilisib for drug-related AEs.

### PK analysis

Blood samples were collected after single and multiple doses of pictilisib, paclitaxel, and letrozole for PK evaluations. Plasma concentrations of pictilisib, paclitaxel, 6α-hydroxy paclitaxel (6α-OH-paclitaxel; cytochrome P450 2C8 [CYP2C8]-formed metabolite of paclitaxel), and letrozole were determined using validated liquid chromatography-tandem mass spectrometry (LC-MS/MS) methods, and PK parameters were estimated using noncompartmental analysis (WinNonlin 6.4; Pharsight, Mountain View, CA USA).

### Outcomes

The primary endpoints for the treatment combinations of pictilisib with letrozole alone, paclitaxel alone, and paclitaxel in combination with bevacizumab or trastuzumab, were safety and tolerability, DLTs, MTD, and identification of a recommended phase II dosing regimen. The secondary endpoints were PK of pictilisib and preliminary antitumor activity (ORR, duration of response [DoR], and PFS). Exploratory objectives included exploration of the potential relationship between PI3K pathway alterations and antitumor activity, as well as identification of the potential role of polymorphisms in drug metabolism enzyme and transporter genes in the PK disposition, and/or response to pictilisib, standard-of-care chemotherapy regimens, or antiestrogen agents.

### Biomarker assessments

Mutational analysis of *PIK3CA* was performed using RT-PCR assays as previously described [30]. Nucleotide substitutions in the amino acids E542 (K), E545 (A, G, D, or K), Q546 (E, K, L, or R), and H1047 (L, R, or Y) or the wild-type alleles were detected. PTEN expression was examined with immunohistochemistry as previously described (clone 138G6; Cell Signaling Technology, Danvers, MA, USA), and an H-score was assigned to each sample on the basis of the percentage of cells staining at four different levels of intensity (0, 1+, 2+, or 3+) [31].

### Statistical methods

Final analysis was performed on cumulative clinical data collected until the last patient’s last visit. The efficacy-evaluable population, which was the basis for ORR analysis, was defined as treated patients with baseline measurable disease and at least one postbaseline tumor assessment, or discontinuation of the study due to disease progression or death within 30 days of treatment initiation. All analyses were based on the safety-evaluable population, which was defined as all enrolled patients who received any dose of pictilisib. This study was designed not with regard to explicit power and type I error considerations, but to obtain preliminary safety and PK information in this patient population. The data cutoff for all analyses was December 1, 2015.

## Results

### Patient characteristics

**Fig. 1:**
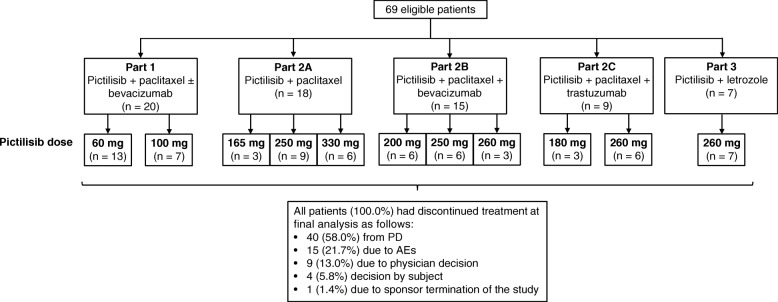
Participant flow diagram. *AE* Adverse event, *PD* Progressive disease

Overall, 69 patients were enrolled in the study (August 2009 to December 2015), with 20 patients in part 1 (pictilisib + paclitaxel ± bevacizumab), 18 patients in part 2A (pictilisib + paclitaxel), 15 patients in part 2B (pictilisib + paclitaxel + bevacizumab), 9 patients in part 2C (pictilisib + paclitaxel + trastuzumab), and 7 patients in part 3 (pictilisib + letrozole) (Fig. 1). At final analysis, all patients had discontinued study treatment because of an AE (21.7%), progressive disease (58.0%), physician decision (13.0%), patient decision (5.8%), or sponsor termination of the study (1.4%). Baseline characteristics were well balanced among treatment groups (Table 1). The median age of all patients was 54.0 years (range, 30–76 years), and the majority of patients (71.0%) had hormone receptor-positive disease.

**Table 1:** Baseline demographics and clinical characteristics

Characteristic	Part 1: pictilisib + paclitaxel ± bevacizumab^a^ (*n* = 20)	Part 2A: pictilisib + paclitaxel (*n* = 18)	Part 2B: pictilisib + paclitaxel + bevacizumab (*n* = 15)	Part 2C: pictilisib + paclitaxel + trastuzumab (*n* = 9)	Part 3: pictilisib + letrozole (*n* = 7)	All patients (*N* = 69)
Median age, years (range)	54.0 (41–71)	53.5 (30–76)	49.0 (34–66)	63.0 (42–68)	59.0 (49–69)	54.0 (30–76)
ECOG PS, *n* (%)						
0	14 (70.0)	6 (33.3)	13 (86.7)	5 (55.6)	4 (57.1)	42 (60.9)
1	5 (25.0)	12 (66.7)	2 (13.3)	4 (44.4)	3 (42.9)	26 (37.7)
Unknown	1 (5.0)	(0.0)	(0.0)	(0.0)	(0.0)	1 (1.4)
ER/PR status, *n* (%)						
Positive	13 (65.0)	11 (61.1)	10 (66.7)	8 (88.9)	7 (100.0)	49 (71.0)
Negative	7 (35.0)	6 (33.3)	5 (33.3)	1 (11.1)	(0.0)	19 (27.5)
Unknown	(0.0)	1 (5.6)	(0.0)	(0.0)	(0.0)	1 (1.4)
HER2 status, *n* (%)						
Positive	(0.0)	(0.0)	(0.0)	9 (100.0)	(0.0)	9 (13.0)
Negative	20 (100.00)	18 (100.00)	15 (100.00)	(0.00)	7 (100.00)	60 (87.00)
Prior chemotherapy, *n* (%)						
Neoadjuvant	6 (30.0)	7 (38.9)	5 (33.3)	3 (33.3)	1 (14.3)	22 (31.9)
Adjuvant setting	12 (60.0)	8 (44.4)	7 (46.7)	4 (44.4)	4 (57.1)	35 (50.7)
Metastatic setting	10 (50.0)	9 (50.0)	8 (53.3)	8 (88.9)	(0.0)	35 (50.7)
Prior treatment, *n* (%)						
Taxanes	10 (50.0)	10 (55.6)	8 (53.3)	6 (66.7)	3 (42.9)	37 (53.6)
Anti-HER2 therapies	2 (10.0)	1 (5.6)	(0.0)	9 (100.0)	(0.0)	12 (17.4)
Bevacizumab	1 (5.0)	2 (11.1)	1 (6.7)	(0.0)	1 (14.3)	5 (7.2)
Line of therapy (metastatic setting)						
First	3 (15.0)	5 (27.8)	1 (6.7)	(0.0)	2 (28.6)	11 (15.9)
Second or later^b^	17 (85.0)	13 (72.2)	14 (93.3)	9 (100.0)	5 (71.4)	58 (84.1)

*Abbreviations: ECOG PS* Eastern Cooperative Oncology Group Performance Status, *ER* Estrogen receptor, *HER2* Human epidermal growth factor receptor, *PR* Progesterone receptor

^a^One patient did not receive bevacizumab

^b^Nineteen patients had four to ten prior lines of therapy

### Safety

The safety profile of all dosing regimens examined in this dose-finding trial was assessed. All patients experienced at least one AE (Table 2), and the most common AEs (reported in ≥ 30% of patients) were diarrhea (78.3%), nausea (62.3%), fatigue (59.4%), alopecia (52.2%), rash (50.7%), neutropenia (44.9%), stomatitis (37.7%), vomiting (33.3%), decreased appetite (33.3%), and cough (30.4%). The most common AEs related to any study drug (≥ 15% of patients) were diarrhea (75.4%), nausea (58.0%), fatigue (56.5%), alopecia (52.2%), rash (46.4%), neutropenia (44.9%), and stomatitis (36.2%) (Additional file 1: Table S1). The majority of patients experienced a grade ≥ 3 AE (*n* = 50; 72.5%) (Table 2), and the most common (in at least two patients) were neutropenia (*n* = 19), rash (*n* = 7), peripheral neuropathy (*n* = 4), hypophosphatemia (*n* = 3), decreased lung diffusing capacity (*n* = 3), dyspnea (*n* = 2), hypertension (*n* = 2), diarrhea (*n* = 2), nausea (*n* = 2), pneumonia (*n* = 2), increased blood glucose (*n* = 2), decreased appetite (*n* = 2), pulmonary embolism (*n* = 2), nail disorder (*n* = 2), and deep vein thrombosis (*n* = 2). In addition, most patients (*n* = 43; 62.3%) experienced at least one grade ≥ 3 AE related to any study drug (Additional file 1: Table S1). Overall, serious AEs were reported in 30.4% of patients (*n* = 21) (Table 2 and Additional file 1: Table S2) and those reported in at least two patients included pneumonia (*n* = 2), nausea (*n* = 2), decreased lung diffusing capacity (*n* = 2), and pulmonary embolism (*n* = 2).

**Table 2:** Safety overview (safety population, regardless of causality)

	Part 1: pictilisib + paclitaxel ± bevacizumab^a^		Part 2A: pictilisib + paclitaxel			Part 2B: pictilisib + paclitaxel + bevacizumab			Part 2C: pictilisib + paclitaxel + trastuzumab		Part 3: pictilisib + letrozole	All patients (*N* = 69)
Pictilisib dose *n* (%)	60 mg (*n* = 13)	100 mg (*n* = 7)	165 mg (*n* = 3)	250 mg (*n* = 9)	330 mg (*n* = 6)	200 mg (*n* = 6)	250 mg (*n* = 6)	260 mg (*n* = 3)	180 mg (*n* = 3)	260 mg (*n* = 6)	260 mg (*n* = 7)	
All-grade AEs	13 (100)	7 (100)	3 (100)	9 (100)	6 (100)	6 (100)	6 (100)	3 (100)	3 (100)	6 (100)	7 (100)	69 (100)
Grades 3–4 AEs	8 (61.5)	7 (100)	3 (100)	7 (77.8)	5 (83.3)	3 (50.0)	5 (83.3)	3 (100)	1 (33.3)	3 (50.0)	3 (42.9)	48 (69.6)
Grade 5 AEs	1 (7.7)^b^	0	0	0	0	0	0	0	0	0	1 (14.3)^c^	2 (2.9)
SAEs	3 (23.1)	1 (14.3)	1 (33.3)	5 (55.6)	5 (83.3)	2 (33.3)	0	0	0	1 (16.7)	3 (42.9)	21 (30.4)
DLTs	1 (7.7)	0	0	1 (11.1)	2 (33.3)	0	1 (16.7)	0	0	1 (16.7)	0	6 (8.7)
Study withdrawal due to AE	3 (23.1)	2 (28.6)	0	1 (11.1)	2 (33.3)	0	1 (16.7)	2 (66.7)	0	2 (33.3)	2 (28.6)	15 (21.7)
Pictilisib withdrawal due to AE	4 (30.8)	2 (28.6)	1 (33.3)	1 (11.1)	2 (33.3)	0	1 (16.7)	3 (100)	0	2 (33.3)	2 (28.6)	18 (26.1)
												*n* = 62
Paclitaxel withdrawal due to AE	6 (46.2)	3 (42.9)	2 (66.7)	1 (11.1)	2 (33.3)	0	1 (16.7)	2 (66.7)	1 (33.3)	3 (50.0)	–	21 (33.9)
												*n* = 35
Bevacizumab withdrawal due to AE	6 (46.2)	3 (42.9)	–	–	–	2 (33.3)	1 (16.7)	2 (66.7)	–	–	–	14 (40.0)
												(n = 9)
Trastuzumab withdrawal due to AE	–	–	–	–	–	–	–	–	0	0 2 (33.3)	–	2 (22.2)
Letrozole withdrawal due to AE	–	–	–	–	–	–	–	–	–	–	2 (28.6)	–
Pictilisib dose reduction due to AE	0	0	0	1 (11.1)	1 (16.7)	0	3 (50.0)	1 (33.3)	0	0	0	6 (8.7)
Pictilisib dose interruption due to AE	7 (53.8)	3 (42.9)	2 (66.7)	5 (55.6)	4 (66.7)	4 (66.7)	3 (50.0)	3 (100)	0	4 (66.7)	4 (57.4)	39 (56.5)

*Abbreviations: AE* Adverse event, *DLT* Dose-limiting toxicity, *ECOG PS* Eastern Cooperative Oncology Group Performance Status, *SAE* Serious adverse event

^a^One patient did not receive bevacizumab

^b^Patient had grade 5 left ventricular dysfunction

^c^Patient had a worsened ECOG PS (grade 5)

Fifteen patients (21.7%) had an AE that led to discontinuation of any study drug (Table 2), and the most common were peripheral neuropathy (*n* = 5), decreased lung diffusing capacity (*n* = 4), rash (*n* = 3), neutropenia (*n* = 2), paresthesia (*n* = 2), pulmonary embolism (*n* = 2), deep vein thrombosis (*n* = 2), and hypertension (*n* = 2). In the case of pictilisib, 18 patients (26.1%) experienced AEs leading to withdrawal (Table 2) and those in at least two patients were decreased lung diffusing capacity (*n* = 4), rash (*n* = 3), and deep vein thrombosis (*n* = 2). AEs that led to pictilisib dose reduction included grade 2 neutropenia (*n* = 3) and grades 1 and 3 rash (*n* = 1 and *n* = 2, respectively). Thirty-nine (56.5%) patients had their pictilisib dose interrupted owing to an AE, whereas six patients (8.7%) had their dose reduced (Table 2). Withdrawal of paclitaxel, bevacizumab, and trastuzumab occurred in 21 patients (33.9%), 14 patients (40.0%), and two patients (22.2%), respectively (Table 2).

AEs of special interest included pneumonitis (3 [4.3%] patients), hyperglycemia or increased blood glucose (15 [21.7%] patients), left ventricular dysfunction (1 [1.4%] patient), and decreased carbon monoxide-diffusing capacity (5 [7.2%] patients) (Additional file 1: Table S3). Grade ≥ 3 AEs of special interest in these patients were reported for hyperglycemia or increased blood glucose (4 [5.8%] patients), left ventricular dysfunction (1 [1.4%] patient), and decreased carbon monoxide-diffusing capacity (3 [4.3%] patients) (Additional file 1: Table S3).

Two patients (2.9%) experienced AEs that led to a fatal outcome (Table 2). One patient had grade 5 left ventricular dysfunction, considered by the investigator to be related to pictilisib, bevacizumab, and paclitaxel. The other patient experienced grade 5 worsened ECOG PS, which was considered by the investigator to be related to pictilisib and unrelated to letrozole.

Overall, six patients (8.7%) reported DLTs (Table 2 and Additional file 1: Table S4). In part 1, one DLT was reported with 60 mg pictilisib, whereas none were observed with the 100-mg dose (pictilisib administered on the “21 + 7” dosing schedule and in combination with paclitaxel and bevacizumab). In part 2A, one DLT was observed in a patient treated with 250 mg pictilisib (“5 + 2” dosing schedule and in combination with paclitaxel), whereas there were two DLTs at the next dose level (330 mg pictilisib); thus, the MTD was exceeded. In part 2B, there was one reported DLT in a patient treated with 250 mg pictilisib (“5 + 2” dosing schedule and administered in combination with paclitaxel and bevacizumab), whereas in part 2C a DLT was observed in one patient treated with 260 mg pictilisib (“5 + 2” dosing schedule and administered in combination with paclitaxel plus trastuzumab). There were no DLTs reported in part 3 (260 mg pictilisib administered continuously with letrozole).

The MTD was defined in part 2A as 250 mg pictilisib (“5 + 2” dosing schedule) in combination with paclitaxel and was not established in all other arms. The MTD or maximum administered dose and recommended phase II doses of pictilisib were 100 mg (when administered with paclitaxel and bevacizumab [“21 + 7” dosing schedule]), 250 mg (when administered with paclitaxel or paclitaxel plus bevacizumab [“5 + 2” dosing schedule]), or 260 mg (when administered with paclitaxel and trastuzumab [“5 + 2” dosing schedule] or letrozole [continuous dosing schedule]).

### PK analysis

**Fig. 2:**
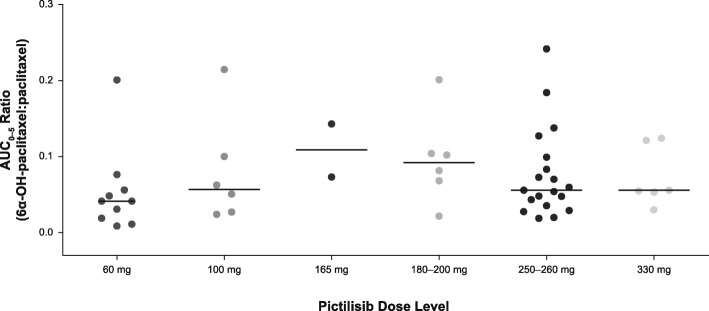
Plasma 6α-OH-paclitaxel/paclitaxel AUC ratio as a function of pictilisib dose. Patients with evaluable 6α-OH-paclitaxel and paclitaxel PK after multiple doses of paclitaxel and pictilisib were pooled across all paclitaxel treatment arms (parts 1 and 2, *n* = 49). *Black lines* represent the median ratio for each dose level, and *dots* represent individual subject ratios. *PK* Pharmacokinetics

In vitro data suggest that pictilisib has a moderate potential to inhibit the CYP2C8-mediated metabolism of paclitaxel to 6α-OH-paclitaxel. In this study, a consistent 6α-OH-paclitaxel:paclitaxel AUC ratio was observed across all pictilisib dose levels (Fig. 2), suggesting there was no drug–drug interaction between pictilisib and paclitaxel. In addition, no differences in the PK of pictilisib or letrozole were observed in any of the treatment combination regimens compared with historical single-agent data (data not shown).

### Clinical activity

**Fig. 3:**
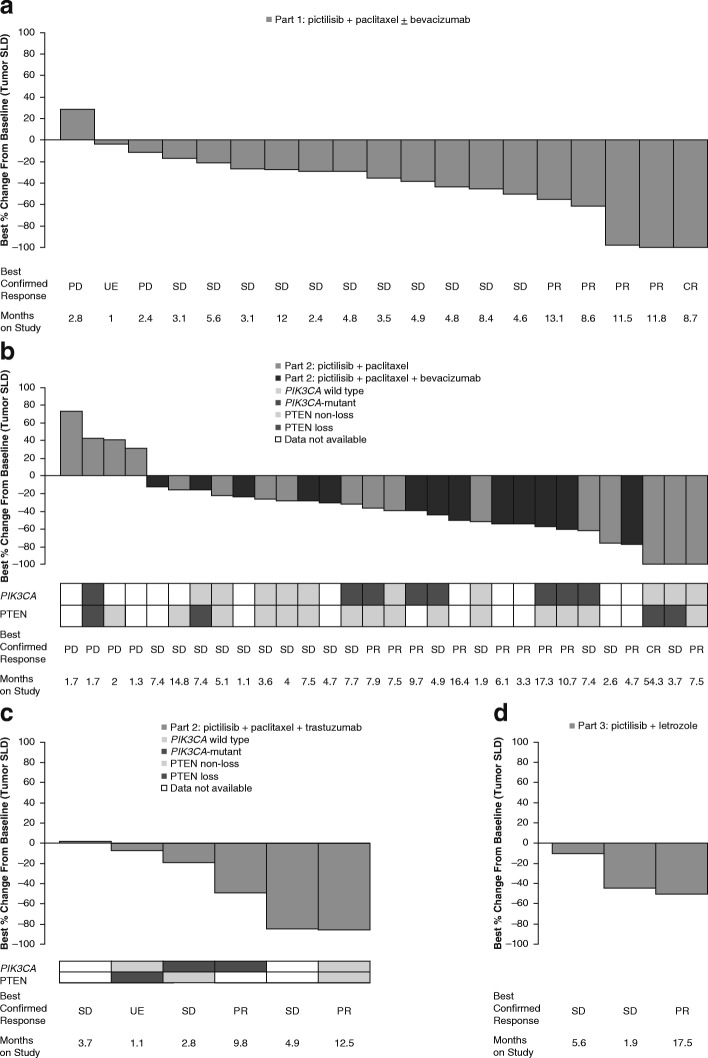
Waterfall plot of maximum percentage changes from baseline in SLD for target lesions. Maximum percentage changes are shown in (**a**) part 1 (pictilisib + paclitaxel ± bevacizumab), (**b**) parts 2A and B (2A: pictilisib + paclitaxel; 2B: pictilisib + paclitaxel + bevacizumab), (**c**) part 2C (pictilisib + paclitaxel + trastuzumab), and (**d**) part 3 (pictilisib + letrozole). PTEN categories were defined as PTEN loss (PTEN H-score ≥ 0 but ≤ 100) or nonloss (PTEN H-score > 100). *CR* Complete response, *MND* Mutation not detectable, *PD* Progressive disease, *PIK3CA* Phosphatidylinositol-45-bisphosphate 3-kinase catalytic subunit-alpha, *PR* Partial response, *PTEN* Phosphatase and tensin homolog, *SD* Stable disease, *SLD* Sum of the longest diameters, *UE* Unevaluable

Fifty-eight (84.1%) patients were included in the efficacy-evaluable population for ORR analysis. Complete responses were observed in two patients (3.4%) overall, in parts 1 (5.3%; paclitaxel + bevacizumab) and 2A (5.9%; pictilisib + paclitaxel) (Table 3). Partial responses were observed in patients treated with pictilisib + paclitaxel ± bevacizumab (part 1; 21.1%), pictilisib + paclitaxel (part 2A; 17.6%), pictilisib + paclitaxel + bevacizumab (part 2B; 53.8%), pictilisib + paclitaxel + trastuzumab (part 2C; 33.3%), and pictilisib + letrozole (part 3; 33.3%) (Table 3). The majority of patients showed signs of tumor shrinkage (Fig. 3a–d).

**Table 3:** Clinical activity in patients with measurable disease at baseline (efficacy-evaluable population)

	Part 1: pictilisib + paclitaxel ± bevacizumab^a^	Part 2A: pictilisib + paclitaxel	Part 2B: pictilisib + paclitaxel + bevacizumab	Part 2C: pictilisib + paclitaxel + trastuzumab	Part 3: pictilisib + letrozole	All patients
ORR						
Best confirmed response, *n* (%)	*n* = 19	*n* = 17	*n* = 13	*n* = 6	*n* = 3	*n* = 58
CR	1 (5.3)	1 (5.9)	(0.0)	(0.0)	(0.0)	2 (3.4)
PR	4 (21.1)	3 (17.6)	7 (53.8)	2 (33.3)	1 (33.3)	17 (29.3)
SD	11 (57.9)	9 (52.9)	6 (46.2)	3 (50.0)	2 (66.7)	31 (53.4)
PD	2 (10.5)	4 (23.5)	(0.0)	(0.0)	(0.0)	6 (10.3)
NE	1 (5.3)	(0.0)	(0.0)	1 (16.7)	(0.0)	2 (3.4)
DoR	*n* = 5	*n* = 4	*n* = 7	*n* = 2	*n* = 1	
Patients with an event, *n* (%)	3 (60.0)	0 (0.0)	5 (71.4)	1 (50.0)	0 (0.0)	–
Median DoR, months	8.9	NE	8.8	NE	NE	–
95% CI	6.47–11.10	NE–NE	4.40–15.34	5.36–NE	NE–NE	–
PFS	*n* = 20	*n* = 18	*n* = 15	*n* = 9	*n* = 7	
Patients with an event, *n* (%)	13 (65.0)	10 (55.6)	10 (66.7)	5 (55.6)	5 (71.4)	–
Median duration of PFS, months	5.8	5.0	7.5	14.8	5.4	–
95% CI	3.52–10.87	3.71–NE	4.60–10.41	3.52–16.62	1.87–NE	–

*Abbreviations: CR* Complete response, *DoR* Duration of response, *NE* Nonevaluable, *PD* Progressive disease, *PFS* Progression-free survival, *PR* Partial response, *SD* Stable disease

a One patient did not receive bevacizumab

**Table 4:** Ethical approval: list of independent ethics committees and institutional review boards

Country	Central ethics committee
United States	Dana Farber Cancer Institute Institutional Review Board
Italy	Comitato Etico Indipendente della Fondazione IRCCS Istituto Nazionale dei Tumori di Milano
Belgium	Commissie Medische Ethiek van de Universitaire Ziekenhuizen KU Leuven
United States	Vanderbilt University Institutional Review Board
United States	University of Illinois College of Medicine at Peoria

The median DoR was 8.9 months (95% CI, 6.47–11.10) among five responders treated with pictilisib + paclitaxel ± bevacizumab (part 1) and 8.8 months (95% CI, 4.40–15.34) among seven responders treated with pictilisib + paclitaxel + bevacizumab (part 2B) (Table 3).

In all treated patients (*N* = 69), median PFS ranged from 5.0 months (95% CI, 3.71–NE) in patients treated with pictilisib + paclitaxel (part 2A; *n* = 18) to 14.8 months (95% CI, 3.52–16.62) in patients treated with pictilisib + paclitaxel + trastuzumab (part 2C; *n* = 9) (Table 3). 

*PIK3CA* mutation status and PTEN expression were evaluated in 24 formalin-fixed, paraffin-embedded tumor samples from parts 2A, 2B and 2C. Four of the 24 samples were evaluable for PTEN only (*n* = 2) or *PIK3CA* only (*n* = 2). PTEN expression was reduced or absent in five of the 22 samples examined, whereas 10 of 22 samples harbored a *PIK3CA* mutation. Among the patients in part 2A and 2B with evaluable tissue and either a *PIK3CA* mutation or PTEN loss, five of 11 (45.5%) had a complete or partial response compared with two of seven (28.6%) patients without these alterations (Fig. 3b).

## Discussion

This phase Ib study evaluated the safety and PK of the pan-PI3K inhibitor pictilisib in combination with paclitaxel, with and without bevacizumab or trastuzumab, or letrozole, in patients with locally recurrent or metastatic breast cancer. At a dose of 260 mg, pictilisib had a manageable safety profile, when combined with paclitaxel, with and without bevacizumab or trastuzumab (“21 + 7” or “5 + 2” dosing schedules) or in combination with 2.5 mg letrozole. The MTD of pictilisib was exceeded in patients treated with 330 mg pictilisib plus paclitaxel. Antitumor activity was observed across all treatment arms. The PK analysis suggested no evidence of a clinical drug–drug interaction between agents in each of the evaluated treatment combination regimens. Taking the potential benefit–risk balance into consideration, 260 mg pictilisib was selected as the recommended phase II dose.

Previous clinical trials of pictilisib have reported inconsistent results. The first-in-human phase I trial of single-agent pictilisib in patients with advanced solid tumors demonstrated evidence of antitumor activity in patients with gastrointestinal stromal tumors, cervical cancer, melanoma, colorectal cancer, cholangiocarcinoma, breast cancer, and ovarian cancer, and it showed that pictilisib was well tolerated [20]. Addition of pictilisib to anastrozole in patients with ER-positive, HER2-negative early breast cancer in the OPPORTUNE study significantly decreased tumor cell proliferation [32]. The randomized phase II PEGGY trial (NCT01740336) did not show any benefit from the addition of pictilisib to paclitaxel in patients with hormone receptor-positive, HER2-negative locally recurrent or metastatic breast cancer [33]. Moreover, a randomized phase II trial (FERGI; NCT01437566) in patients with ER-positive, HER2-negative, endocrine-resistant breast cancer found that the addition of pictilisib to fulvestrant did not significantly improve PFS [34]. Although the phase III BELLE-2 study (NCT01610284) reported modest improvements in median PFS (1.9 months) in patients with hormone receptor-positive metastatic breast cancer treated with the pan-PI3K inhibitor buparlisib (BKM120) in combination with fulvestrant (versus placebo plus fulvestrant) [35], combining a PI3K inhibitor with different therapies is challenging. In both the PEGGY and FERGI studies, efficacy was likely limited by the higher incidence of AEs and dose reductions/discontinuations owing to AEs with pictilisib treatment [33, 34]. As a result, further development of pictilisib by the sponsor is not planned.

Exploratory biomarker analyses in this study showed a numerical difference in ORR between tumors that harbored a *PIK3CA* mutation or had PTEN loss (45.5%) and those without these alterations (28.6%); however, this study was not powered to distinguish antitumor activity between these patient groups, and results were interpreted with caution owing to small patient numbers. Previous studies found little evidence for a link between *PIK3CA* mutations and antitumor activity with pictilisib [32–34].

Although there was evidence of antitumor activity with pictilisib in this patient population, the sample size was limited. Thus, because efficacy was not the primary endpoint and the study was not powered to detect meaningful differences between study arms, the conclusions of the observed antitumor activity are limited.

Targeting the PI3K pathway with isoform-specific inhibitors may decrease dose modifications caused by toxicity. Alpelisib showed some single-agent activity, with a favorable safety profile, in patients with *PIK3CA*-mutant advanced breast cancer [36]. In addition, alpelisib in combination with fulvestrant demonstrated clinical activity in patients with ER-positive, *PIK3CA*-mutated, locally advanced or metastatic breast cancer [37], whereas alpelisib in combination with letrozole was well tolerated, with evidence of clinical activity in patients with ER-positive metastatic breast cancer that was refractory to endocrine therapy [38]. Taselisib, a potent and selective PI3K inhibitor that has greater selectivity for mutant PI3Kα isoforms than wild-type PI3Kα [23, 39, 40], has single-agent activity in tumors with *PIK3CA* mutations [41]. Thus, taselisib is currently being evaluated in combination with fulvestrant in postmenopausal women with ER-positive, HER2-negative, *PIK3CA*-mutant, locally advanced or metastatic breast cancer (ClinicalTrials.gov identifier NCT02340221) [42].

## Conclusions

The combination of pictilisib with paclitaxel, with and without bevacizumab or trastuzumab, or letrozole, in this phase Ib study had an acceptable safety profile with manageable toxicities, with evidence of antitumor activity in patients with locally recurrent or metastatic breast cancer. The effect of pictilisib in combination with paclitaxel supported further investigation in a randomized clinical study.

## Additional file

Additional file 1: **Table S1.** All-grade AEs related to any study drug occurring in ≥ 15% of all patients and corresponding grade ≥ 3 AEs. **Table S2.** SAEs related to any study drug (regardless of causality). **Table S3.** AEs and grade ≥ 3 AEs of special interest (regardless of causality). **Table S4.** Summary of DLTs observed during the study. (DOCX 66 kb)

## Abbreviations

6α-OH-paclitaxel: 6α-Hydroxy paclitaxel
AE: Adverse event
ALP: Alkaline phosphatase
CR: Complete response
DLT: Dose-limiting toxicities
DoR: Duration of response
ECOG PS: Eastern Cooperative Oncology Group Performance Status
ER: Estrogen receptor
HER2: Human epidermal growth factor receptor 2
MND: Mutation not detectable
MTD: Maximum tolerated dose
mTOR: Mammalian target of rapamycin
NE: Nonevaluable
ORR: Objective response rate
PD: Progressive disease
PFS: Progression-free survival
PI3K: Phosphatidylinositol 3-kinase
PIK3CA: Phosphatidylinositol-4,5-bisphosphate 3-kinase catalytic subunit alpha
PK: Pharmacokinetics
PR: Partial response
PTEN: Phosphatase and tensin homolog
RECIST: Response Evaluation Criteria In Solid Tumors
SAE: Serious adverse event
SD: Stable disease
SLD: Sum of the longest diameters
UE: Unevaluable
ULN: Upper limit of normal
