# Supplementary material for: A phase Ib study of pictilisib (GDC-0941) in combination with paclitaxel, with and without bevacizumab or trastuzumab, and with letrozole in advanced breast cancer
Source: Breast Cancer Res. 2018 Sep 5;20:109. doi: 10.1186/s13058-018-1015-x (PMC6125885; doi:10.1186/s13058-018-1015-x)
Supplement: Supplementary file 1 — Table S1. All-grade AEs related to any study drug occurring in ≥ 15% of all patients and corresponding grade ≥ 3 AEs. Table S2. SAEs related to any study drug (regardless of causality). Table S3. AEs and grade ≥ 3 AEs of special interest (regardless of causality). Table S4. Summary of DLTs observed during the study. (DOCX 66 kb) [file 13058_2018_1015_MOESM1_ESM.docx]

**Additional file 1**

**Table S1** All-grade AEs related to any study drug occurring in ≥15% of all patients and corresponding grade ≥3 AEs

|  | Part 1: pictilisib + paclitaxel ± bevacizumab  (n = 20)^a^ | | Part 2A: pictilisib + paclitaxel  (n = 18) | | Part 2B: pictilisib + paclitaxel + bevacizumab  (n = 15) | | Part 2C: pictilisib + paclitaxel + trastuzumab  (n = 9) | | Part 3: pictilisib + letrozole  (n = 7) | | All patients  (N = 69) | |
| --- | --- | --- | --- | --- | --- | --- | --- | --- | --- | --- | --- | --- |
|  | All-grade | Grade ≥3 AEs | All-grade | Grade ≥3 AEs | All-grade | Grade ≥3 AEs | All-grade | Grade ≥3 AEs | All-grade | Grade ≥3 AEs | All-grade | Grade ≥3 AEs |
| All | 20 (100.0) | 16 (80.0) | 18 (100.0) | 11 (61.1) | 15 (100.0) | 10 (66.7) | 9 (100.0) | 3 (33.3) | 7 (100.0) | 3 (42.9) | 69 (100.0) | 43 (62.3) |
| Diarrhea | 14 (70.0) | 1 (5.0) | 17 (94.4) | 1 (5.6) | 11 (73.3) | 0 | 5 (55.6) | 0 | 5 (71.4) | 0 | 52 (75.4) | 2 (2.9) |
| Nausea | 11 (55.0) | 0 | 9 (50.0) | 0 | 7 (46.7) | 0 | 7 (77.8) | 0 | 6 (85.7) | 2 (28.6) | 40 (58.0) | 2 (2.9) |
| Fatigue | 13 (65.0) | 0 | 9 (50.0) | 0 | 11 (73.3) | 0 | 2 (22.2) | 0 | 4 (57.1) | 1 (14.3) | 39 (56.5) | 1 (1.4) |
| Alopecia | 13 (65.0) | – | 11 (61.1) | – | 7 (46.7) | – | 5 (55.6) | – | 0 | – | 36 (52.2) | 0 |
| Rash | 7 (35.0) | 0 | 11 (61.1) | 2 (11.1) | 7 (46.7) | 4 (26.7) | 6 (66.7) | 1 (11.1) | 1 (14.3) | 0 | 32 (46.4) | 7 (10.1) |
| Neutropenia | 13 (65.0) | 9 (45.0) | 10 (55.6) | 6 (33.3) | 6 (40.0) | 4 (26.7) | 2 (22.2) | 0 | 0 | 0 | 31 (44.9) | 19 (27.5) |
| Stomatitis | 9 (45.0) | 0 | 8 (44.4) | 0 | 5 (33.3) | 0 | 2 (22.2) | 0 | 1 (14.3) | 0 | 25 (36.2) | 0 |
| Decreased appetite | 2 (10.0) | 0 | 8 (44.4) | 1 (5.6) | 4 (26.7) | 0 | 1 (11.1) | 0 | 5 (71.4) | 1 (14.3) | 20 (29.0) | 2 (2.9) |
| Vomiting | 1 (5.0) | 0 | 8 (44.4) | 0 | 3 (20.0) | 0 | 4 (44.4) | 0 | 4 (57.1) | 1 (14.3) | 20 (29.0) | 1 (1.4) |
| Nail disorder | 8 (40.0) | 2 (10.0) | 5 (27.8) | 0 | 2 (13.3) | 0 | 4 (44.4) | 0 | 0 | 0 | 19 (27.5) | 2 (2.9) |
| Dysgeusia | 2 (10.0) | 0 | 6 (33.3) | 0 | 6 (40.0) | 0 | 1 (11.1) | 0 | 3 (42.9) | 0 | 18 (26.1) | 0 |
| Peripheral sensory neuropathy | 8 (40.0) | 1 (5.0) | 6 (33.3) | 0 | 2 (13.3) | 0 | 1 (11.1) | 0 | 0 | 0 | 17 (24.6) | 1 (1.4) |
| Epistaxis | 6 (30.0) | 0 | 1 (5.6) | 0 | 8 (53.3) | 0 | 1 (11.1) | 0 | 0 | 0 | 16 (23.2) | 0 |
| Myalgia | 4 (20.0) | 0 | 6 (33.3) | 0 | 2 (13.3) | 0 | 3 (33.3) | 0 | 0 | 0 | 15 (21.7) | 0 |
| Paresthesia | 2 (10.0) | 0 | 6 (33.3) | 0 | 2 (13.3) | 0 | 4 (44.4) | 0 | 0 | 0 | 14 (20.3) | 0 |
| Asthenia | 2 (10.0) | 0 | 3 (16.7) | 1 (5.6) | 2 (13.3) | 0 | 5 (55.6) | 0 | 0 | 0 | 12 (17.4) | 1 (1.4) |
| Headache | 3 (15.0) | 0 | 1 (5.6) | 0 | 4 (26.7) | 0 | 4 (44.4) | 0 | 0 | 0 | 12 (17.4) | 0 |
| Constipation | 2 (10.0) | 0 | 1 (5.6) | 0 | 5 (33.3) | 0 | 2 (22.2) | 0 | 1 (14.3) | 0 | 11 (15.9) | 0 |

AE, adverse event.

**Table S2** SAEs related to any study drug (regardless of causality)

|  | Part 1: pictilisib + paclitaxel ± bevacizumab | | Part 2A: pictilisib +  paclitaxel | | | | Part 2B: pictilisib +  paclitaxel + bevacizumab | | | Part 2C: pictilisib + paclitaxel + trastuzumab | | | Part 3: pictilisib + letrozole | All patients  (N = 69) |
| --- | --- | --- | --- | --- | --- | --- | --- | --- | --- | --- | --- | --- | --- | --- |
| Pictilisib dose  n (%) | 60 mg  (n = 13)^a^ | 100 mg  (n = 7) | 165 mg  (n = 3) | 250 mg  (n = 9) | 330 mg  (n = 6) | 200 mg  (n = 6) | | 250 mg  (n = 6) | 260 mg  (n = 3) | | 180 mg (n = 3) | 260 mg  (n = 6) | 260 mg (n = 7) |  |
| All^b^ | 3 (23.1) | 1 (14.3) | 1 (33.3) | 5 (55.6) | 5 (83.3) | 2 (33.3) | | 0 | 0 | | 0 | 1 (16.7) | 3 (42.9) | 21 (30.4) |
| Pneumonia | 0 | 0 | 1 (33.3) | 0 | 1 (16.7) | 0 | | 0 | 0 | | 0 | 0 | 0 | 2 (2.9) |
| Nausea | 0 | 0 | 0 | 1 (11.1) | 1 (16.7) | 0 | | 0 | 0 | | 0 | 0 | 0 | 2 (2.9) |
| Carbon monoxide diffusing capacity decreased | 1 (7.7) | 0 | 0 | 0 | 1 (16.7) | 0 | | 0 | 0 | | 0 | 0 | 0 | 2 (2.9) |
| Pulmonary embolism | 1 (7.7) | 0 | 0 | 0 | 0 | 1 (16.7) | | 0 | 0 | | 0 | 0 | 0 | 2 (2.9) |

^a^ One patient did not receive bevacizumab. ^b^ All SAEs, regardless of causality.

SAE, serious adverse event.

**Table S3** AEs and grade ≥3 AEs of special interest (regardless of causality)

|  | Part 1: pictilisib + paclitaxel ± bevacizumab | | Part 2A: pictilisib +  paclitaxel | | | Part 2B: pictilisib +  paclitaxel + bevacizumab | | | Part 2C: pictilisib + paclitaxel + trastuzumab | | Part 3: pictilisib + letrozole | All patients  (N = 69) |
| --- | --- | --- | --- | --- | --- | --- | --- | --- | --- | --- | --- | --- |
| Pictilisib dose  n (%) | 60 mg  (n = 13)^a^ | 100 mg  (n = 7) | 165 mg  (n = 3) | 250 mg  (n = 9) | 330 mg  (n = 6) | 200 mg  (n = 6) | 250 mg  (n = 6) | 260 mg  (n = 3) | 180 mg (n = 3) | 260 mg  (n = 6) | 260 mg (n = 7) |  |
| **AEs** |  |  |  |  |  |  |  |  |  |  |  |  |
| Pneumonitis | 0 | 0 | 0 | 0 | 0 | 1 (16.7) | 1 (16.7) | 0 | 1 (33.3) | 0 | 0 | 3 (4.3) |
| Blood glucose increased | 2 (15.4) | 0 | 0 | 0 | 0 | 0 | 0 | 0 | 0 | 0 | 0 | 2 (2.9) |
| Hyperglycemia | 0 | 2 (28.6) | 0 | 3 (33.3) | 0 | 1 (16.7) | 1 (16.7) | 0 | 2 (66.7) | 0 | 4 (57.1) | 13 (18.8) |
| Left ventricular dysfunction | 1 (7.7) | 0 | 0 | 0 | 0 | 0 | 0 | 0 | 0 | 0 | 0 | 1 (1.4) |
| Carbon monoxide diffusing capacity decreased | 1 (7.7) | 1 (14.3) | 0 | 0 | 2 (33.3) | 0 | 0 | 1 (33.3) | 0 | 0 | 0 | 5 (7.2) |
| **Grade ≥3 AEs** |  |  |  |  |  |  |  |  |  |  |  |  |
| Pneumonitis | 0 | 0 | 0 | 0 | 0 | 0 | 0 | 0 | 0 | 0 | 0 | 0 |
| Blood glucose increased | 2 (15.4) | 0 | 0 | 0 | 0 | 0 | 0 | 0 | 0 | 0 | 0 | 2 (2.9) |
| Hyperglycemia | 0 | 0 | 0 | 2 (22.2) | 0 | 0 | 0 | 0 | 0 | 0 | 0 | 2 (2.9) |
| Left ventricular dysfunction | 1 (7.7)^b^ | 0 | 0 | 0 | 0 | 0 | 0 | 0 | 0 | 0 | 0 | 1 (1.4) |
| Carbon monoxide diffusing capacity decreased | 1 (7.7) | 1 (14.3) | 0 | 0 | 1 (16.7) | 0 | 0 | 0 | 0 | 0 | 0 | 3 (4.3) |

^a^ One patient did not receive bevacizumab. ^b^ Patient had grade 5 left ventricular dysfunction.

AE, adverse event.

**Table S4** Summary of DLTs observed during the study

| Treatment regimen and dose level | Patients with DLT | DLT experienced | Grade |
| --- | --- | --- | --- |
| 60 mg pictilisib + paclitaxel + bevacizumab (Part 1) | 1 | Deep vein thrombosis | 3 |
| 250 mg pictilisib + paclitaxel (Part 2A) | 1 | Febrile neutropenia | 3 |
| 330 mg pictilisib + paclitaxel (Part 2A) | 2 | Rash | 3 |
|  |  | Bacteremia | 3 |
| 250 mg pictilisib + paclitaxel + bevacizumab (Part 2B) | 1 | Rash | 3 |
| 260 mg pictilisib + paclitaxel + trastuzumab (Part 2C) | 1 | Rash | 3 |

DLT, dose-limiting toxicities.
